# Supplementary material for: Relative Validity of a Short 15-Item Food Frequency Questionnaire Measuring Dietary Quality, by the Diet History Method
Source: Nutrients. 2021 Oct 24;13(11):3754. doi: 10.3390/nu13113754 (PMC8622557; doi:10.3390/nu13113754)
Supplement: Supplementary file 1 [file nutrients-13-03754-s001.zip › nutrients-1364553-supplementary.pdf]

Title: Relative validity of a short 15-item food frequency questionnaire measuring dietary quality, by the diet history method

Supplementary material

Food frequency questionnaire.

Supplementary table S1. Food groups.

Food Frequency Questionnaire

ID number -- **ID**

*Select **one option** for each question - indicate what <u>you usually do</u>!*

1. How often do you eat vegetables (fresh, frozen, or cooked)?

- ☐ Twice a day or more often (1)
- ☐ Once a day (2)
- ☐ A few times a week (3)
- ☐ Once a week or less (4)

☐ **K 162**

2. How often do you eat fruit and/or berries (fresh, frozen, preserved, juice, etc.)?

- ☐ Twice a day or more often (1)
- ☐ Once a day (2)
- ☐ A few times a week (3)
- ☐ Once a week or less (4)

☐ **K 163**

3. How often do you eat nuts (almonds, peanuts, hazelnuts, pistachio nuts, pine nuts, walnuts, cashews)?

- ☐ Twice a day or more often (1)
- ☐ Once a day (2)
- ☐ A few times a week (3)
- ☐ Once a week or less (4)

☐ **K 164**

4. How often do you eat fish or shellfish?

- ☐ Three times a week or more often (1)
- ☐ Twice a week (2)
- ☐ Once a week (3)
- ☐ A few times a month or less (4)

☐ **K 165**

5. How often do you eat red meat (beef, pork, or game)?

- ☐ Three times a week or more often (1)
- ☐ Twice a week (2)
- ☐ Once a week (3)
- ☐ A few times a month or less (4)

☐ **K 166**

6. How often do you eat white meat (poultry e.g. chicken)?

- ☐ Three times a week or more (1)
- ☐ Twice a week (2)
- ☐ Once a week (3)
- ☐ A few times a month or less (4)

☐ **K 167**

7. How often do you eat buns/cakes, chocolate/sweets, crisps or soda/juice?

- ☐ Twice a day or more often (1)
- ☐ Once a day (2)
- ☐ A few times a week (3)
- ☐ Once a week or less (4)

☐ **K 168**

8. How often do you eat breakfast?

- ☐ Every day (1)
- ☐ Almost every day (2)
- ☐ A few times a week (3)
- ☐ Once a week or less (4)

☐ **K 169**

9. How many slices/pieces of bread do you eat per day in total?

- ☐ I don't eat bread (00)

Number of slices/pieces ___________ ☐☐ **K 170**

What type(s) of bread do you eat?

- ☐ White bread (1)
- ☐ Whole wheat bread (labeled as high-fiber, low fat, and low sugar) (2)
- ☐ Crispbread (3)
- ☐ Other, please specify (4) ____
- ☐ Combinations of the above (5)

☐ **K 171**

10. How often do you drink/eat milk, sour milk and/or yoghurt?

- ☐ Twice a day or more often (1)
- ☐ Once a day (2)
- ☐ A few times a week (3)
- ☐ Once a week or less (4)

☐ **K 172**

11. What type of milk, sour milk and/or yoghurt do you usually drink/eat?

- ☐ Whole / full fat (3%) (1)
- ☐ Semi-skimmed / reduced fat (1.5%) (2)
- ☐ Skimmed / low fat (0.5%) or non-fat (0.1%) (3)

☐ **K 173**

12. What kind of spread do you usually use on sandwiches?
Select *one* option, the one you usually use?

- ☐ Butter (1)
- ☐ Spread containing 75% fat (2)
- ☐ Margarine (3)
- ☐ Spread made with seed and plant oils containing 70% fat (4)
- ☐ Low-fat margarine containing 30-40% fat (5)
- ☐ Margarine with plant sterols (6)
- ☐ I don't use spread on sandwiches (7)
- ☐ I don't know (8) ☐ **K 174**

13. What kind of fat do you usually use for cooking at home?
Select *one* option - the one you usually use!

- ☐ Butter (1)
- ☐ Margarine containing 60-80% fat (2)
- ☐ Cooking margarine (3)
- ☐ Margarine made with seed and plant oils (4)
- ☐ Liquid margarine (5)
- ☐ Vegetable oil, e.g. rapeseed oil, olive oil, corn oil, sunflower oil (6)
- ☐ I don't use fat in cooking (7)
- ☐ I don't know (8) ☐ **K 175**

14. Do you usually add salt to your food?

- ☐ No (0)
- ☐ Yes, sometimes (1)
- ☐ Yes, often (2)
- ☐ Yes, I always add salt before I taste the food (3) ☐ **K 176**

15. Do you consciously avoid salty foods?

- ☐ No (0)
- ☐ Yes (1) ☐ **K 177**

16. Have you followed any diet during the past year?

- ☐ No (0)
- ☐ Yes (1) ☐ **K 178**

If yes, which one(s)?

	All the time (1)	In periods (2)	Currently (3)		
LCHF (low carbohydrate, high fat) or The Atkins Diet	☐	☐	☐	☐	**K 179**
The GI diet	☐	☐	☐	☐	**K 180**
Weight Watchers	☐	☐	☐	☐	**K 181**
The Mediterranean Diet	☐	☐	☐	☐	**K 182**
Low calorie meal replacement powders/bars	☐	☐	☐	☐	**K 183**
**Other,** state which one: …………………………………………..	☐	☐	☐	☐	**K 184**

Supplementary table S1. Food groups according to the Diet history (DH) and those applicable to the Food Frequency Questionnaire (FFQ)

Number	Item in FFQ	Food group DH	Registered food intake DH
1	4	Fish and shellfish/seafood	Fish and seafood and dishes, caviar and spawn
2	5	Meat and processed meat	Meat, processed meat, tripe, offal and blood meals and dishes
3	6	Poultry	Poultry and poultry dishes
4	Not applicable (Na)	Eggs	Egg and egg dishes
5	Na	Potatoes	Potatoes and dishes
6	1	Vegetables and pulses	Vegetables, root vegetables, mushrooms, pulses and dishes
7	2	Fruits and berries	Fruits and berries including canned or dried
8	11, 12	Keyhole* milk products	Keyhole milk products; fat content ≤0.7 % and for flavoured products limit for sugars ≤9%
9	11, 12	Non-Keyhole milk products	Non-Keyhole milk products, including sweet milk drinks
10	Na	Cream and crème fraîche	Cream, sour cream and crème fraîche
11	Na	Cheese	Cheese and cheese dishes
12	Na	Fast food	Burgers, doner-kebab, falafel, pizza, hot dogs, taco meals

13	Na	Pasta, rice and food grain	Pasta, rice, food grains and dishes
14	9, 10	Bread refined	Bread refined, soft bread and crisp bread <5 % fibre
15	9, 10	Bread fibre-rich	Bread fibre-rich, soft bread and crisp bread >5 % fibre
16	Na	Cereals	Breakfast cereals (hot and cold), porridge and gruel, starch oatmeal
17	7	Savoury bakery	Savoury bakery, pasty, savoury pancakes and crêpes, sandwich layer cake, taco shells, crackers, savoury pie
18	7	Sweet bakery	Buns, cookies, cakes
19	7	Desserts	Sweet pie, crumble, chocolate mousse, cheesecake, sweet soups, ice cream, etc.
20	Na	Sweet condiments	Sugar, syrup, honey and sweeteners, jam, marmalade, sweet cacao powder
21	7	Sweets, candy and chocolate	Sweets, candy, candy bars, chocolate
22	1	Salads	Greek salad, chicken salad, pasta salads, etc.
23	Na	Soups	Soups, broth
24	Na	Sauces, dressings and condiment	Sauces, dressings, aioli, coleslaw, ronnaise salads, ketchup, HP sauce
25	Na	Substitute products	Soya milk, oat milk, coconut milk, tofu, Quorn and soya products
26	13, 14	Margarine	Table margarine and soft margarine for cooking, including butter-based
27	13, 14	Butter	Butter, lard
28	13, 14	Vegetable oil	Vegetable oils
29	7	Snacks	Crisps, popcorn, cheese doodles, tortilla chips, etc.

30	3	Nuts and seeds	Nuts and seeds including coconut flakes, tahini, peanut butter, etc.
31	7	Juice	Fruit and vegetable juices including pure fruit shots
32	Na	Coffee	Coffee
33	Na	Tea	Tea
34	Na	Soda	Soda, lemonade, sports and energy drinks, non-alcoholic cider, regular and light
35	Na	Alcoholic beverages	Wine, beer, alcoholic cider, drinks, spirits, liqueur ≥1 % alcohol

*Keyhole is the Swedish National Food Agency-labelling scheme, which guides healthy food choices. For milk and yogurt to meet the criteria for the Keyhole, fat content has to be limited to a maximum of 0·7%, and for flavoured products there is an additional limit for sugars: a maximum of 9 %.

[a] Excluded food items: Puddings, custard, cheesecake, meals, cottage cheese, pickled vegetables and olives, coconut milk, coconut fat, soups, tomato sauce
